# The Importance of Biochemical Parameters, Immunonutritional Status, and Social Support for Quality of Life in Chronic Hemodialysis Patients

**DOI:** 10.3390/medicina60111751

**Published:** 2024-10-24

**Authors:** Batric Babovic, Natasa Belada Babovic, Filip Tomovic, Snezana Radovanovic, Mladen Debeljevic, Dusan Mustur, Olgica Mihaljevic

**Affiliations:** 1 Department of Nephrology, Clinical Center of Montenegro, 81000 Podgorica, Montenegro; babovicbatric@gmail.com (B.B.); tomovic.filip28@gmail.com (F.T.); 2 Department of Cardiology, Clinical Center of Montenegro, 81000 Podgorica, Montenegro; beladan@t-com.me; 3 Department of Social Medicine, Faculty of Medical Sciences, University of Kragujevac, 34000 Kragujevac, Serbia; jovanarad@yahoo.com; 4 Department of Neurology, Clinical Center of Montenegro, 81000 Podgorica, Montenegro; mladen.debeljevic@t-com.me; 5 Faculty of Medicine, University of Montenegro, 81000 Podgorica, Montenegro; dusanm@ucg.ac.me; 6 Department of Pathophysiology, Faculty of Medical Sciences, University of Kragujevac, 34000 Kragujevac, Serbia

**Keywords:** chronic kidney disease, immunonutrition, hemodialysis, quality of life, social support, systemic inflammation, uremia

## Abstract

*Background and Objectives*: Chronic kidney disease (CKD) is a growing public health problem and one of the leading causes of premature death worldwide. The progressive nature of CKD is associated with serious complications that can reduce the quality of life in CKD patients. Additional factors that can worsen well-being include dialysis treatment, malnutrition, inflammation, and lack of social support. The aim of our study was to analyze the quality of life of CKD patients undergoing hemodialysis and its association with certain biochemical and immunonutritional parameters, as well as with social support. *Materials and Methods*: This research was conducted as a cross-sectional study that included 170 patients, divided into two groups: a group of patients undergoing hemodialysis (HD group) (n = 85), and a control group of non-hemodialysis patients (group with CKD stage 3–4) (n = 85). The Health-Related Quality of Life (HRQoL) score was used to assess the quality of life of the study population. Measurement of biochemical and immunonutritional parameters was also performed in all patients. The Oslo-3 Social Support Scale (OSSS-3) was used to analyze social support. *Results*: The HRQoL score was significantly lower in HD patients compared to patients with CKD stage 3–4 (0.701 ± 0.137 vs. 0.832 ± 0.122, *p* < 0.001). It declined significantly as the concentrations of urea (β = −0.347, *p* < 0.001), creatinine (β = −0.699, *p* = 0.005), uric acid (β = −0.184, *p* = 0.016), β2-microglobulin (β = −0.432, *p* < 0.001), and parathormone (β = −0.209, *p* = 0.006) increased in HD patients. In addition to uremic toxins, an increase in glucose (β = −0.278, *p* = 0.010) and triglyceride (β = −0.354, *p* = 0.001) concentrations was associated with poor HRQoL in patients with CKD stage 3–4. There was a significant connection between the Hemoglobin, Albumin, Lymphocyte, and Platelet (HALP) score and HRQoL in HD patients (β = 0.229, *p* = 0.035). Additionally, C-reactive protein (β = −0.361, *p* < 0.001) and neutrophil-to-lymphocyte ratio (β = −0.288, *p* < 0.001), as markers of systemic inflammation, directly affected HRQoL in HD patients. In both study groups, perceived social support positively influenced the HRQoL scores (β = 0.192, *p* = 0.012 for hemodialysis; β = 0.225, *p* = 0.038 for non-hemodialysis). *Conclusions*: There is a decline in HRQoL in chronic hemodialysis patients, significantly affected by certain biochemical and immunonutritional parameters, along with perceived social support.

## 1. Introduction

Chronic kidney disease (CKD) is a progressive decline in kidney function, recognized as a growing public health problem. The Global Burden of Disease Study has shown that the prevalence of CKD is 10–15%, affecting more than 840 million individuals worldwide [1]. It is one of the leading causes of years of life lost, associated with an increased all-age mortality rate [2].

The progressive nature of CKD is linked to serious complications that could lead to a decrease in overall quality of life, especially in advanced stages of the disease. Quality of life is a critical outcome measure for CKD patients, influenced by disease-related manifestations and the complex procedure of hemodialysis with frequent hospital visits. Changes in physical (reduced mobility, usual activity, and self-care), psychological (presence of pain, depression, and anxiety), and social functions (reduced interactions with others, including family members) in CKD patients affect all aspects of multidomain determinants of well-being. In that sense, dialysis therapy represents a particularly heavy burden in an individual’s daily life, due to physical limitations and psychological stress [3]. The presence of disease symptoms, inability to meet basic needs independently, withdrawal from social life, and social isolation make people with CKD a particularly vulnerable group, with significantly reduced quality of life. Many studies have pointed out a low quality of life in dialysis patients, highlighting the challenges of accepting the nature of chronic disease as part of their self-identity [4,5,6].

Additional aggravating factors implicated in CKD progression and well-being are inflammation and malnutrition [7]. Chronic low-grade inflammation in CKD is multifactorial, associated with reduced renal clearance of pro-inflammatory mediators [8], comorbidities [9], endogenous intoxication with uremic toxins [10], and an anti-inflammatory cytokine genotype [11]. Chronic inflammation and malnutrition may cause immunosuppression, increasing the risk of infection and mortality in patients with chronic conditions [12].

According to the available literature data, new biomarkers of immunonutritional status are important in patients with chronic conditions. These include the Hemoglobin, Albumin, Lymphocyte, and Platelet (HALP) score and the Prognostic Nutritional Index (PNI) (obtained from serum albumin concentration and total blood lymphocyte count) [7,12]. These scores reflect a patient’s overall health status by integrating routinely determined laboratory parameters. Until now, their prognostic utility has been estimated in numerous conditions, such as cancers [13], coronary disease [14], autoimmune diseases [15], and acute and chronic kidney dysfunction [7,16]. It is believed that the incorporation of certain nutritional and inflammatory markers may provide a better model for monitoring CKD progression and patients’ quality of life. Previous investigations have highlighted the importance of systemic inflammation markers in predicting the course and outcome of CKD patients undergoing dialysis. Thus, higher concentrations of C-reactive protein (CRP) [17], as well as elevated values of the neutrophil-to-lymphocyte ratio (NLR) and platelet-to-lymphocyte ratio (PLR), have been observed in hemodialysis patients [18]. The NLR and PLR have been shown to significantly correlate with traditional markers of inflammation and pro-inflammatory cytokines like tumor necrosis factor α (TNF-α) in advanced CKD stages [19].

On the other hand, the impact of social support is crucial for patients with CKD undergoing dialysis in accepting their disease. Adequate social support can positively influence the disease course, improving both mental and physical health [20].

Given that well-being is considered crucial for CKD patient-centered outcomes—more so than longevity—our study aimed to analyze the quality of life in chronic hemodialysis patients and its relationship with certain biochemical parameters, immunonutritional parameters, and perceived social support.

## 2. Materials and Methods

### 2.1. Study Design and Participants

This research was conducted as a single-center cross-sectional study that included 170 patients with CKD treated in the Nephrology Clinic of the Clinical Center of Montenegro, Podgorica. The KDIGO criteria were used to confirm the diagnosis of CKD [21], while the stage of the disease was determined based on the value of glomerular filtration rate (GFR) obtained by the Modification of Diet in Renal Disease (MDRD) Equation [22]. The study population was divided into two groups: a group of patients undergoing chronic hemodialysis (HD group) (n = 85), and a control group of non-hemodialysis patients (group with CKD stage 3–4) (n = 85). The inclusion criteria for participation in the study were as follows: (1) established diagnosis of CKD and appropriate therapeutic modality for at least 6 months until the moment of inclusion in the study, (2) age > 18 years, and (3) signed informed consent for participation in the study. The study did not include individuals with (1) acute infections (up to one month before inclusion in the study), (2) previously diagnosed and/or treated autoimmune diseases and chronic inflammatory diseases, (3) malignant diseases, (4) the presence of cognitive impairments and functional limitations, or (5) pregnancy. A thrice-weekly hemodialysis regimen for four hours was performed in all HD patients. This was administered via a forearm arteriovenous fistula with an extracorporeal blood flow rate of 250–300 mL/min using a Fresenius 5008 hollow-fiber dialyzer and an FX 80 polysulfone dialysis membrane (Fresenius Medical Care, Bad Homburg, Germany). Bicarbonate solution with a standard bicarbonate concentration of 35 mmol/L was used as the dialysis fluid. Blood samples from all patients were taken once: just before starting the first HD session of the week in HD patients, and just before starting the medical examination for non-hemodialysis patients (and upon obtaining consent for participation in the study).

This study was conducted in accordance with the Declaration of Helsinki and was approved by the local ethics committee (number 03/01-29521/1). Informed written consent was obtained from all patients after a thorough explanation of the study.

### 2.2. Data Collection

Data about demographic and clinical characteristics of the patients were obtained directly from the patients, during physical examination, as well as from their medical records. We calculated body mass index (BMI) in all patients as the person’s weight in kilograms divided by the square of height in meters.

Hemodialysis efficiency (Kt/V) was calculated by the dialysis machine software as the ratio of urea clearance (K) multiplied by dialysis time (t) to the volume of water (V) in the body. Additionally, the total clearance received during each treatment (Kt) was also determined, considering that volume could affect the estimation of the minimum amount of hemodialysis, especially in malnourished patients.

### 2.3. Evaluation of Quality of Life

A standardized 15-dimensional (15D) questionnaire and Health-Related Quality of Life (HRQoL) score were used to assess the quality of life of the study population (consent obtained from the author of the questionnaire, Prof. Dr. Harri Sintonen from the University of Helsinki) [23]. This questionnaire includes 15 dimensions: mobility, vision, hearing, breathing, sleep, nutrition, speech (communication), voiding, usual activities, mental function, discomfort, depression, anxiety, vitality, and sexual activity. Each dimension is analyzed and divided into 5 levels, with the first level corresponding to normal function and the last to very impaired function. After testing each dimension, the HRQoL score was calculated using the 15D evaluation algorithm. The total score values range from 0 to 1, with a higher value indicating a better quality of life.

### 2.4. Measurement of Biochemical Parameters

Standard accepted methods were used for the analysis of biochemical parameters at the Center for Clinical Laboratory Diagnostics of the Clinical Center of Montenegro. Serum concentrations of urea, creatinine, uric acid, total proteins, albumins, cholesterol, triglycerides, glucose, electrolytes (sodium (Na^+^), potassium (K^+^), calcium (Ca^2+^), and phosphates (PO_4_^3−^)), and intact parathyroid hormone (iPTH) were measured on a Beckman Coulter AU5800 (Beckman Coulter Inc. Brea, CA, USA). β2-Macroglobulin concentration was evaluated by the Atellica NEPH 630 analyzer (Siemens Healthineers, Forchheim, Germany) and N Latex β2-Microglobulin reagent.

### 2.5. Parameters of Inflammation and Immunonutritional Status

Serum concentrations of CRP were measured on a Beckman Coulter AU5800 analyzer (Beckman Coulter Inc. Brea, CA, USA), while certain indices were calculated from the absolute number of neutrophil leukocytes and lymphocytes (NLR), or from the number of platelets and lymphocytes (PLR). The counts of leukocytes, leukocyte subtypes, and platelets were obtained by the automated DxH 800 Hematology Analyzer (Beckman Coulter, Inc., Brea, CA, USA).

The Prognostic Nutritional Index (PNI), proposed by Buzby et al. [24], was calculated using the following formula: (10 × serum albumin [g/dL]) + (0.005 × total lymphocyte count [k/mm^3^]).

The Hemoglobin, Albumin, Lymphocyte, and Platelet (HALP) score, developed by Chen et al. [25] to predict prognosis in gastric carcinoma, was calculated by hemoglobin (g/L) × albumin (g/L) × lymphocytes (/L)/platelets (/L).

### 2.6. Evaluation of Social Support

The Oslo-3 Social Support Scale (OSSS-3) was used to analyze social support [26]. It is a 3-item self-reported measure of the level of social support related to the number of close confidants, the sense of concern from other people, and the relationships with neighbors in terms of practical help. The OSSS-3 score is obtained by adding up the respondents’ answers (scoring in the first question from 1 (“no person”) to 4 (“5+ people”), from 1 (“not at all interested”) to 5 (“very interested”) in the second question, and from 1 (“very difficult”) to 5 (“very easy”) in the last question. The total OSSS-3 score ranges from 3 to 14, and it is interpreted as follows: poor social support (3–8), moderate social support (9–11), and strong social support (12–14).

### 2.7. Statistical Analysis

The data obtained were processed using the standard program package SPSS, version 22.0. Continuous variables are presented as the mean value ± standard deviation, while categorical variables are presented as a percentage of respondents. Depending on the distribution, differences in the mean values of continuous variables were compared using the independent-samples T test or the Mann–Whitney U test. Univariate and multivariate linear regression tests were used to analyze the relationships between dependent and independent variables and determine the predictive significance of biochemical parameters, immunonutritional parameters, and social support on the study subjects’ quality of life. Values of *p* ≤ 0.05 were considered statistically significant.

## 3. Results

### 3.1. Demographic and Clinical Characteristics of the Study Population

**Figure 1:**
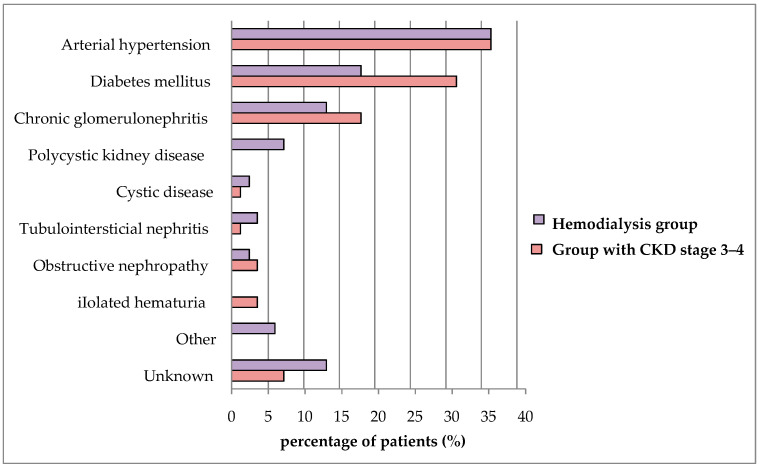
The distribution of the study population according to the main cause of chronic kidney disease.

A total of 170 patients with CKD were included in the study. Among them, there were 89 (52.4%) males and 81 (47.6%) females, with an average age of 60.14 ± 13.08 years. In terms of treatment, 85 patients underwent hemodialysis (HD group) (56.5% males and 43.5% females), while 85 patients did not undergo hemodialysis (group with CKD stage 3–4) (48.2% males and 51.8% females). Among the non-hemodialysis patients, 35 (41.2%) were in stage “G3a”, 29 (34.1%) in stage “G3b”, and 21 (24.7%) in stage “G4” of CKD. The mean age of the HD patients was 58.76 ± 12.62 years (range 22–81 years), while the mean age of patients from the control group was 61.52 ± 13.47 years (range 22–81 years). There was no statistically significant difference in the average age of patients in the two analyzed groups (Mann–Whitney test, *p* = 0.127). As for the main causes of CKD, arterial hypertension and diabetes mellitus were the most prevalent in both hemodialysis patients (hypertension in 35.3% of subjects and DM in 17.6% of subjects) and CKD stage 3–4 patients (hypertension in 35.3% of subjects and DM in 30.6% of subjects) (Figure 1).

The average value of GFR obtained by the MDRD Equation was 4.96 ± 1.87 mL/min/1.73 m^2^ in HD patients (range 2.60–14.40 mL/min/1.73 m^2^), while it was 40.22 ± 12.62 mL/min/1.73 m^2^ in the group of patients with CKD stage 3–4 (range 17.50–59.10 mL/min/1.73 m^2^) (Mann–Whitney test, *p* < 0.001). The duration of HD varied from 10 to 300 months, with a median of 32 months (IQR 26.50). Analysis of HD treatment adequacy indicated a mean Kt/V value of 1.26 ± 1.13 (range 0.90–1.60) and a mean value of Kt 53.21 ± 8.09 L (range 39.58–73.92 L).

### 3.2. Health-Related Quality of Life in the Study Population

**Figure 2:**
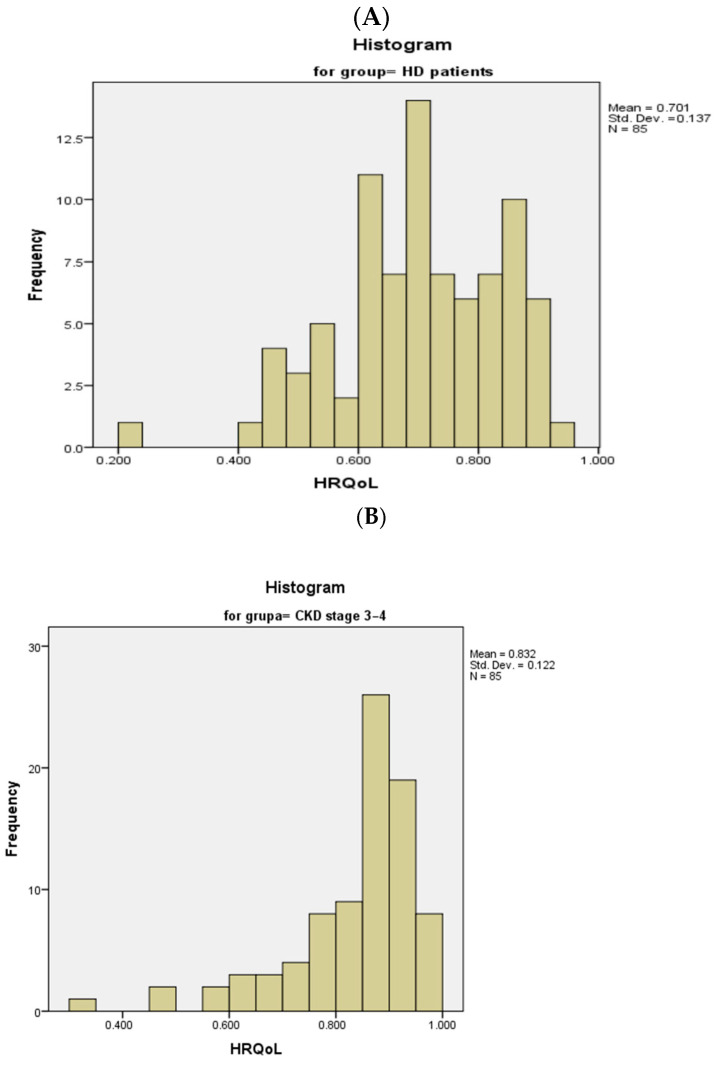
Histograms of HRQoL score distribution in hemodialysis patients (**A**) and CKD stage 3–4 patients (**B**).

Using the 15D evaluation algorithm developed by Harri Sintonen, we evaluated the Health-Related Quality of Life (HRQoL) of our respondents. The results showed significantly lower values of HRQoL score in HD patients compared to patients with CKD stage 3–4 (Mann–Whitney test, *p* < 0.001). The mean HRQoL score was 0.701 ± 0.137 (range 0.232–0.926; median 0.701) in patients undergoing hemodialysis, while it was 0.832 ± 0.122 (range 0.309–0.966; median 0.859) in non-hemodialysis patients (Figure 2).

### 3.3. Biochemical, Immunonutritional, and Inflammation Parameters in the Study Population

The concentrations of analyzed biochemical, immunonutritional, and inflammation parameters in the study population are shown in Table 1. A statistically significant difference in all biochemical parameters between the two groups of patients was observed. The concentrations of urea (*p* < 0.001), creatinine (*p* < 0.001), uric acid (*p* = 0.029), β2-macroglobulin (*p* < 0.001), potassium (*p* < 0.001), phosphates (*p* = 0.018), and parathyroid hormone (*p* < 0.001) were significantly higher in HD patients compared to patients with CKD stage 3–4.

In terms of immunonutritional status, patients undergoing hemodialysis had a significantly lower mean BMI value (*p* = 0.018), as well as lower concentrations of total cholesterol (*p* < 0.001), triglycerides (*p* < 0.001), and glucose (*p* = 0.004). Similarly, the concentration of hemoglobin (*p* < 0.001), hematocrit value (*p* = 0.038), and blood iron concentration (*p* = 0.001) were significantly decreased in dialysis patients compared to control subjects. Unlike the erythrocytes (*p* < 0.001), there were no statistically significant differences in the average numbers of white blood cells (*p* = 0.648), neutrophils (*p* = 0.525), lymphocytes (*p* = 0.480), or platelets (*p* = 0.067). There was a significantly lower concentration of albumin in hemodialysis patients (*p* < 0.001), leading to an expected reduction in PNI in this group (*p* < 0.001). Although the HALP values were slightly lower in HD patients compared to patients with CKD stage 3–4, these differences did not reach statistical significance (*p* = 0.252).

In terms of systemic inflammation parameters, HD patients had significantly higher CRP levels (*p* < 0.001) and NLR index scores (*p* = 0.006) than patients with CKD stage 3–4. There was no significant difference in the average values of PLR between the two groups of study subjects (*p* = 0.705).

### 3.4. Social Support in the Study Population

**Figure 3:**
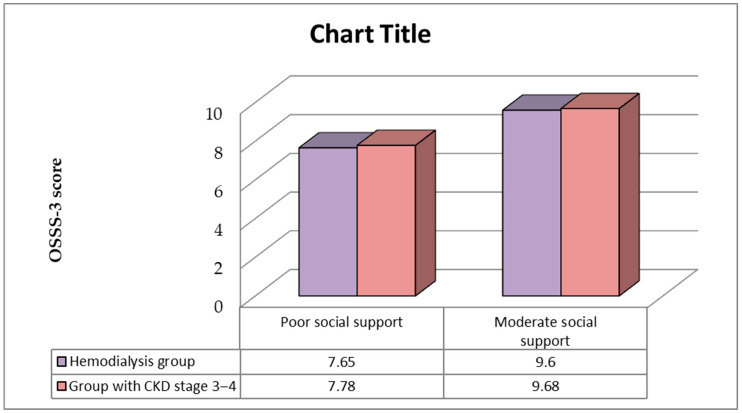
OSSS-3 scores in the study population.

Analysis of the degree of social support by the Oslo-3 Social Support Scale (OSSS-3) indicated that the mean OSSS-3 score in HD patients was 8.87 ± 1.19 (min 5, max 11), while the mean OSSS-3 score in patients with CKD stage 3–4 was 9.08 ± 1.09 (min 7, max 11) (Mann–Whitney test, *p* = 0.307). Among HD patients, 32 (37.6%) had poor social support and 53 (62.4%) had moderate social support. Among patients with CKD stage 3–4, 27 (31.8%) had poor social support and 58 (68.2%) had moderate social support. There were no patients with good social support. The relationship of OSSS-3 values between the two groups of patients is shown in Figure 3.

### 3.5. The Association of Biochemical Parameters, Immunonutritional Parameters, and the Degree of Social Support with Quality of Life in the Study Population

Finally, we examined the predictive significance of all independent variables for HRQoL to determine the association of biochemical parameters, immunonutritional parameters, and the degree of social support with the quality of life of patients with CKD (Table 2).

Regression analysis revealed that HRQoL scores declined significantly as the concentrations of urea (β = −0.347, *p* < 0.001), creatinine (β = −0.699, *p* = 0.005), uric acid (β = −0.184, *p* = 0.016), β2-microglobulin (β = −0.432, *p* < 0.001), and iPTH (β = −0.209, *p* = 0.006) increased in HD patients. There was also a negative impact of creatinine (β = −0.476, *p* < 0.001), uric acid (β = −0.277, *p* = 0.010), β2-microglobulin (β = −0.224, *p* = 0.040), and iPTH (β = −0.314, *p* = 0.003) on the quality of life in patients with CKD stage 3–4. An increase in glucose (β = −0.278, *p* = 0.010) and triglyceride (β = −0.354, *p* = 0.001) concentrations was associated with poor HRQoL in patients with CKD stage 3–4. In terms of immunonutritional status, we found a direct connection between HALP and HRQoL score in HD patients, where lower values of the prognostic index corresponded to worse quality of life (β = 0.229, *p* = 0.035). Malnutrition expressed through hemoglobin (β = 0.297, *p* < 0.001) and blood iron (β = 0.203, *p* = 0.008) concentrations directly affected the quality of life in HD patients. There was a significant negative impact of systemic inflammation on HRQoL scores in HD patients: CRP (β = −0.361, *p* < 0.001) and NLR (β = −0.288, *p* < 0.001). In both study groups, perceived social support positively influenced the quality of life (β = 0.192, *p* = 0.012 for hemodialysis; β = 0.225, *p* = 0.038 for non-hemodialysis).

Multivariate regression analysis was used to evaluate the variables that were statistically significant in the univariate model. The influence of the following parameters on the quality of life of patients with CKD was determined: the concentration of creatinine (β = −0.354, *p* = 0.012), NLR (β = −0.162, *p* = 0.023), and social support score (β = 0.119, *p* = 0.039) in HD patients; the concentrations of creatinine (β = −0.329, *p* = 0.002), iPTH (β = −0.212, *p* = 0.032), and triglycerides (β = −0.235, *p* = 0.020) in patients with CKD stage 3–4 (Table 3).

## 4. Discussion

The aim of this study was to analyze the quality of life in chronic hemodialysis patients and its potential connection with certain biochemical and immunonutritional parameters, as well as the degree of social support. For the first time, we evaluated the relationship of prognostic indices (HALP and PNI) with quality of life in HD patients, which have mainly been studied as predictors of mortality so far. Indirectly, we aimed to indicate the impact of laboratory parameters and perceived social support on the outcome of the disease from the perspective of the patients’ well-being.

Our main results are as follows: (1) HRQoL scores were significantly lower in HD patients compared to patients with CKD stage 3–4. (2) There was a negative impact of uremic toxins, malnutrition, and systemic inflammation on HRQoL scores in HD patients, while perceived social support positively influenced the quality of life in HD patients. (3) Accordingly, the concentrations of uremic toxins, CRP, and the NLR index were significantly higher in HD patients compared to patients with CKD stage 3–4. (4) HD patients had a worse nutritional status compared to control subjects; significantly lower mean values of BMI, total cholesterol, triglycerides, and glucose were estimated. Additionally, the concentration of hemoglobin, hematocrit value, blood iron concentration, and RBC count were significantly decreased in HD patients compared to patients with CKD stage 3–4. (5) The overall prognosis expressed through the value of PNI was better in non-hemodialysis patients.

Quality of life is a valuable multidimensional instrument for assessing the outcome of a disease, influenced by a number of factors of both endogenous and exogenous nature. The World Health Organization (WHO) defines quality of life as “individuals’ perceptions of their position in life in the context of the culture and value systems in which they live and in relation to their goals, expectations, standards and concerns” [26]. In the other words, quality of life represents a wide range of human experiences based on overall well-being and related to personal expectations.

The association between HRQoL and the progression of CKD has been well documented. CKD is a global health problem, also known as the “global killer in plain sight”, expected to become the fifth-leading cause of premature death by 2040 [2]. Symptoms associated with CKD’s progression not only negatively affect the quality of life of patients but also intensify other characteristic symptoms. Fatigue or psychological problems faced by people with CKD (e.g., mood fluctuations, fear of disability and death) may be associated with changes in appetite, sleep disturbances, and impairments in physical and social functioning [27].

Unlike previous studies that focused on medical and socio-economic factors when analyzing HRQoL in CKD, we aimed to assess the overall well-being of our patients in relation to their levels of uremia, inflammation, and malnutrition. Considering the loss of support structures and relationships often experienced by CKD patients, we also explored the connection between HRQoL and perceived social support. All analyzed parameters were compared between two groups of subjects: the group of HD patients, and the group of patients with CKD stage 3–4.

CKD is characterized by chronic systemic inflammation, known as uremic hypercytokinemia, especially in the advanced stages of the disease. Factors contributing to chronic inflammation in CKD include reduced renal clearance of cytokines, presence of comorbidities, decreased metabolism of advanced glycosylation products, back-diffusion of contaminated dialysate, infection of vascular access, or penetration of bacterial degradation products through the dialysis membrane [8,9,10,28]. Additionally, the genotype of anti-inflammatory cytokines may play a role in the progression of chronic inflammation in dialysis patients [11]. Previous studies have suggested that elevated pro-inflammatory mediators in CKD patients could be linked to cognitive decline, emotional health issues, and poor social functioning, with uremic toxins potentially playing a significant role in these conditions. The presence of pro-inflammatory cytokines has also been detected in specific nuclei of the central nervous system in a rat model of CKD [29].

Our study revealed higher concentrations of uremic toxins (urea, creatinine, uric acid, β2-microglobulin, iPTH) and inflammatory markers (CRP and NLR) in HD patients compared to patients with CKD stage 3–4. Uremic toxins, systemic inflammation, and malnutrition had a negative impact on HRQoL scores in HD patients. Although PTH levels were relatively low in our patients, we found a significant relationship between PTH and HRQoL scores. Previous research indicated that extra-high or -low PTH could both affect the quality of life and mortality in CKD patients [30,31]. Additionally, low PTH has been associated with low-grade inflammation, which has been shown to have a negative impact on individuals’ cognitive performance, along with slightly poorer self-reported physical functioning and quality of life [32,33].

Conversely, the concentrations of total cholesterol, triglycerides, and glucose were significantly higher in the group of patients with CKD stage 3–4 compared to the rest of the study population. Elevated triglycerides and glucose levels were linked to poor HRQoL, suggesting that low-grade inflammation may play a role in the metabolic changes observed in patients with CKD stage 3–4. Insulin resistance, a key feature of type 2 diabetes, is an early metabolic change in CKD patients and may be influenced by chronic inflammation, lipid dysmetabolism, and vitamin D deficiency with consequent hyperparathyroidism [34]. Uremic toxins also contribute to metabolic disturbances by inhibiting enzymes involved in lipid metabolism, leading to a cycle of low-grade inflammation known as meta-inflammation [35].

However, data published so far indicate a significant influence of nutritional derangements on the course of CKD and its complications [36]. Malnutrition in CKD occurs as a result of poor nutrient intake and negative energy balance associated with uremic syndrome and systemic chronic inflammation, and it is known as an independent prognostic factor for major clinical outcomes, including survival. Additional factors involved in the pathogenesis of malnutrition are disorders of protein, carbohydrate, and lipid metabolism, oxidative stress, hormonal alterations (the action of thyroid hormone, growth hormone, and insulin), metabolic acidosis, and dialysis-related problems (increased protein breakdown as a consequence of the activation of certain metabolic pathways by the HD procedure itself). Pro-inflammatory mediators appear to have a direct influence on the satiety center, but they could also cause an increase in skeletal muscle protein breakdown [37].

Therefore, we determined a statistically significantly lower average value of BMI in HD patients, who also showed reduced concentrations of hemoglobin, blood iron, and RBCs compared to non-hemodialysis patients. Anemia is a frequent companion of CKD that significantly contributes to increases in morbidity and mortality, as well as to a decrease in the quality of life of patients with CKD. Stauffer et al. estimated a twice-as-frequent occurrence of anemia in patients with CKD compared to the general population, which showed a positive trend with the progression of the disease [38]. It seems that the severity of anemia positively correlates with the degree of JGF reduction. The mechanisms responsible for anemia in CKD are numerous. Low erythropoietin levels are assumed to be the primary cause of anemia, but also decline in iron absorption, bleeding and blood loss during HD sessions, interleukin 6 (IL-6)-mediated hepcidin release by the liver, impairment of erythropoiesis induced by uremic toxins, and deficiency of vitamin B12 and/or folic acid [39]. Pro-inflammatory cytokines, such as interleukin 1 (IL-1) and TNF-α, might cause the suppression of bone marrow and could inhibit hypoxia-induced production of erythropoietin. They can also affect iron metabolism by promoting the uptake of transferrin-bound iron into macrophages via the transferrin receptor, thereby limiting the availability of iron in CKD [40]. Data from the literature indicate the multiple impacts of anemia on reducing the quality of life in CKD patients, some of which imply a reduction in functional capacity, work productivity, and social activity due to fatigue, weakness, shortness of breath, headaches, and feelings of sadness or depression, as well as a potential worsening of existing complications of the disease, especially cardiovascular complications [41]. Anemia is known to be associated with angina pectoris, development of left ventricular hypertrophy, and congestive heart failure.

Moreover, it was estimated that our HD patients had slightly lower HALP values and significantly lower PNI values compared to the control subjects. In addition to albumin and lymphocytes, which are important components of the inflammatory reaction, HALP takes into account the values of hemoglobin and platelets (due to possible disorders of hemostasis induced by inflammation in CKD). Low concentrations of hemoglobin and hypoalbuminemia are actually manifestations of malnutrition. Our results are in agreement with the findings of Antar and colleagues [12], who demonstrated that participants who underwent dialysis treatment in the past 12 months had a significantly lower HALP score compared to those without dialysis treatment. Zhang et al. [42] also established a negative association between HALP and the risk of all-cause death in HD patients, suggesting that HALP before dialysis might be a reliable indicator of a poor prognosis. When it comes to PNI, recent research has confirmed the clinical utility of this score, indicating a significant association of higher PNI levels with lower mortality in patients with CKD [7]. Unlike previous research, which was mostly based on the analysis of the prognostic significance of HALP and PNI scores in different types of tumors [43], our study evaluated HALP and PNI in CKD subjects and revealed their impact on the quality of life of HD patients.

Facing the aggressive nature of the disease and questioning the value of life and living after the introduction of dialysis therapy undoubtedly reduce well-being in CKD [44]. As expected, the quality of life of HD patients in our study was significantly worse compared to the quality of life of patients with CKD stage 3–4. We established a decline in HRQoL scores in HD patients with higher levels of uremic inflammation and malnutrition. Combined with the HALP score, certain determinants of malnutrition and inflammation directly affected the quality of life in our HD patients. This is the first study to evaluate the predictive value of nutritional status and systemic inflammation for the quality of life in Montenegrin HD patients, which were previously studied exclusively in terms of their effects on mortality. Previously, Gerasimoula indicated that sociodemographic and clinical characteristics could influence the quality of life of HD patients [4]. Some investigators elucidated that a higher educational level is associated with a better quality of life in HD patients [45], probably because education allows for a deep understanding of the disease. Also, it seems that patients’ adaptation to new life circumstances over time is responsible for less pronounced but not negligible changes in the psychological sphere. Conversely, changes in the physical domain of quality of life seem to be more pronounced and are the result of functional damage due to deterioration of kidney function [46].

On the other hand, social support has proven to be extremely important in the process of accepting the disease, confirming that a higher level of perceived support results in better well-being in HD patients. Previous investigations have shown a positive relationship between social support and CKD patient outcomes, indicating that perceived social support improves treatment adherence and quality of life in HD patients [47,48]. Consistent with this, perceived social support positively influenced the quality of life in both study groups included in our research.

In fact, our results suggest that both immunonutritional status and social support may contribute to the advancement of quality of life in HD patients.

Despite certain limitations (such as a cross-sectional study with one-time sampling, single-center data, etc.), our research indicates that good clinical practice in the treatment of dialysis patients requires an individual approach to ensure a satisfactory quality of life.

A deeper understanding of the factors that affect the quality of life in hemodialysis patients, along with their personal needs, could improve the outcome of a disease that is among the leading causes of death worldwide. Monitoring individuals’ experiences, disease symptomatology, and appropriate laboratory findings are necessary in the process of creating strategies to improve care measures and enhance the quality of life in hemodialysis patients.

## 5. Conclusions

The quality of life is significantly reduced in chronic hemodialysis patients compared to patients with CKD stage 3–4. Uremic toxins, malnutrition, and systemic inflammation have a negative impact on HRQoL scores, while perceived social support positively affects HRQoL in HD patients.

## Figures and Tables

**Table 1:** The concentrations of biochemical, immunonutritional, and inflammation parameters in the study population.

Parameters	Reference Range	Hemodialysis Patients n = 85	Patients with CKD Stage 3–4 n = 85	*p* *
		X ± SD		
**Urea (mmol/L)**	2.5–7.5	24.66 ± 5.89	11.49 ± 8.04	<0.001
**Creatinine (μmol/L)**	62–115 M 44–88 F	916.75 ± 238.88	149.16 ± 54.39	<0.001
**Uric acid (μmol/L)**	211–452 M 147–400 F	379.71 ± 80.21	346.42 ± 72.33	0.029
**β2-Macroglobulin (mg/L)**	1.22–2.46	25.72 ± 5.64	19.04 ± 5.61	<0.001
**Na^+^ (mmol/L)**	137–147	136.16 ± 3.95	139.18 ± 2.61	<0.001
**K^+^ (mmol/L)**	3.5–5.3	5.72 ± 0.99	4.56 ± 0.59	<0.001
**Ca^2+^ (mmol/L)**	2.15–2.50	2.25 ± 0.22	2.43 ± 0.18	<0.001
**PO_4_^3−^ (mmol/L)**	0.74–1.52	1.91 ± 0.62	1.68 ± 0.59	0.018
**iPTH (pmol/L)**	1.6–7.2	28.63 ± 34.41	13.84 ± 25.89	<0.001
**BMI (kg/m^2^)**	18.5–24.9	24.17 ± 4.51	25.26 ± 3.47	0.018
**Total proteins (g/L)**	60–83	69.82 ± 6.42	69.40 ± 5.33	0.682
**Albumins (g/L)**	35–52	37.06 ± 4.11	40.49 ± 5.47	<0.001
**Total cholesterol (mmol/L)**	<5.2	3.68 ± 0.93	5.14 ± 1.93	<0.001
**Triglycerides (mmol/L)**	<1.7	1.58 ± 0.83	2.53 ± 0.92	<0.001
**Glucose (mmol/L)**	3.8–6.1	5.81 ± 2.56	6.04 ± 1.77	0.004
**Hemoglobin (g/L)**	138–175 M 110–157 F	105.37 ± 15.81	126.01 ± 22.06	<0.001
**Hematocrit (L/L)**	0.415–0.530 M 0.356–0.470 F	0.340 ± 0.049	0.355 ± 0.046	0.038
**Blood iron (μmol/L)**	11–28 M 6.6–26 F	10.40 ± 4.72	12.23 ± 2.94	0.001
**RBCs (×10^12^/L)**	4.34–5.72 M 3.86–5.08 F	3.69 ± 0.85	4.15 ± 0.67	<0.001
**WBCs (×10^9^/L)**	3.7–10	6.43 ± 1.88	6.27 ± 1.68	0.648
**Neutrophils (×10^9^/L)**	2.10–6.50	4.08 ± 1.57	3.85 ± 1.09	0.525
**Lymphocytes (×10^9^/L)**	1.20–3.40	1.26 ± 0.59	1.28 ± 0.52	0.480
**Platelets (×10^9^/L)**	135–450	209.95 ± 73.35	237.96 ± 84,72	0.067
**HALP**	/	28.56 ± 20.27	29.42 ± 17.20	0.252
**PNI**	/	376.87 ± 41.46	411.32 ± 55.17	<0.001
**CRP (mg/L)**	<5	4.43 ± 3.07	1.13 ± 1.16	<0.001
**NLR**	/	2.82 ± 1.27	2.28 ± 0.90	0.006
**PLR**	/	202.95 ± 86.64	198.36 ± 63.20	0.705

* Statistical significance determined by independent-samples T test/Mann–Whitney U test. Na^+^, sodium; K^+^, potassium; Ca^2+^, calcium; PO_4_^3−^, phosphates; iPTH, intact parathyroid hormone; BMI, body mass index; RBCs, red blood cells; WBCs, white blood cells; HALP, Hemoglobin, Albumin, Lymphocyte, and Platelet score; PNI, Prognostic Nutritional Index; CRP, C-reactive protein; NLR, neutrophil-to-lymphocyte ratio; PLR, platelet-to-lymphocyte ratio.

**Table 2:** Univariate regression analysis of the influence of biochemical parameters, immunonutritional parameters, and social support on the quality of life (HRQoL score) of CKD patients.

	Hemodialysis Patients			Patients with CKD Stage 3–4		
	HRQoL Score					
	β	95%CI for β	p *	β	95%CI for β	p *
**Urea (per 1 mmol/L)**	−0.347	−0.564–0.090	<0.001	−0.029	−0.275–0.230	0.790
**Creatinine (per 1 μmol/L)**	−0.699	−0.933–−0.213	0.005	−0.476	−0.772–0.036	<0.001
**Uric acid (per 1 μmol/L)**	−0.184	−0.627–−0.271	0.016	−0.277	−0.546–−0.010	0.010
**β** **2-Microglobulin** **(per 1 mg/L)**	−0.432	−0.701–−0.033	<0.001	−0.224	−0.591–0.045	0.040
**Na^+^ (per 1 mmol/L)**	0.105	−0.089–0.298	0.337	0.083	−0.163–0.340	0.451
**K^+^ (per 1 mmol/L)**	−0.072	−0.363–0.146	0.511	−0.156	−0.439–0.113	0.154
**Ca^2+^ (per 1 mmol/L)**	−0.039	−0.393–0.316	0.724	−0.147	−0.296–0.016	0.154
**PO_4_^3−^ (per 1 mmol/L)**	−0.165	−0.537–0.206	0.132	−0.090	−0.342–0.146	0.413
**iPTH (per 1 pmol/L)**	−0.209	−0.578–0.178	0.006	−0.314	−0.546–0.076	0.003
**BMI (per 1 kg/m^2^)**	−0.039	−0.515–0.445	0.724	−0.029	−0.415–0.363	0.794
**Total proteins (per 1 g/L)**	0.012	−0.283–0.312	0.872	0.155	−0.098–0.394	0.157
**Albumins (per 1 g/L)**	0.056	−0.191–0.314	0.510	0.124	0.097–0.332	0.258
**Total cholesterols** **(per 1 mmol/L)**	−0.146	−0.418–0.124	0.058	−0.173	−0.485–−0.099	0.114
**Triglycerides** **(per 1 mmol/L)**	−0.132	−0.366–0.080	0.085	−0.354	−0.562–−0.137	0.001
**Glucose (per 1 mmol/L)**	−0.058	−0.430–0.308	0.453	−0.278	−0.598–−0.048	0.010
**Hemoglobin (per 1 g/L)**	0.297	−0.072–0.585	<0.001	0.051	−0.338–0.444	0.640
**Hematocrit (per 1 L/L)**	0.125	−0.227–0.480	0.105	0.017	−0.296–0.318	0.880
**Blood iron (per 1 μmol/L)**	0.203	−0.150–0.542	0.008	0.054	−0.102–0.356	0.626
**RBCs (per 1 × 10^12^/L)**	0.114	−0.187–0.432	0.139	0.099	−0.111–0.306	0.367
**WBCs (per 1 × 10^9^/L)**	−0.027	−0.453–0.389	0.730	0.110	−0.129–0.323	0.315
**Neutrophils (per 1 × 10^9^/L)**	−0.004	−0.420–0.428	0.959	0.120	−0.154–0.388	0.273
**Lymphocytes (per 1 × 10^9^/L)**	0.102	−0.292–0.498	0.185	−0.014	−0.311–0.283	0.899
**Platelets (per 1 × 10^9^/L)**	0.141	−0.165–0.442	0.067	0.097	−0.203–0.402	0.377
**HALP (per 1 unit)**	0.229	−0.234–0.678	0.035	0.077	−0.246–389	0.486
**PNI (per 1 unit)**	0.076	−0.472–0.634	0.326	0.122	−0.174–0.420	0.267
**CRP (per 1 mg/L)**	−0.361	−0.621–0.093	<0.001	−0.182	−0.499–0.239	0.095
**NLR (per 1 unit)**	−0.288	−0.917–0.321	<0.001	−0.079	−0.481–0.341	0.470
**PLR (per 1 unit)**	−0.078	−0.251–0.053	0.314	−0.035	−0.324–0.250	0.748
**OSSS-3 score (per 1)**	0.192	0.090–0.386	0.012	0.225	0.071–0.428	0.038

* Statistical significance determined by univariate regression test; *β*, regression coefficient; 95%CI, confidence interval. Na^+^, sodium; K^+^, potassium; Ca^2+^, calcium; PO_4_^3−^, phosphates; iPTH, intact parathyroid hormone; BMI, body mass index; RBCs, red blood cells; WBCs, white blood cells; HALP, Hemoglobin, Albumin, Lymphocyte, and Platelet score; PNI, Prognostic Nutritional Index; CRP, C-reactive protein; NLR, neutrophil-to-lymphocyte ratio; PLR, platelet-to-lymphocyte ratio; OSSS-3, Oslo-3 Social Support Scale.

**Table 3:** Multivariate regression analysis of the influence of certain biochemical parameters, immunonutritional parameters, and social support on the quality of life (HRQoL score) of CKD patients.

	**HRQoL Score**			
		**β**	**95%CI for** **β**	p *
**Hemodialysis** **patients**	Creatinine (per 1 μmol/L)	−0.354	−0.660–−0.148	0.012
	NLR (per 1 unit)	−0.162	−0.387–0.014	0.023
	OSSS-3 score (per 1 unit)	0.119	0.031–0.441	0.039
**Patients with** **CKD stage 3–4**	Creatinine (per 1 μmol/L)	−0.329	−0.531–0.060	0.002
	iPTH (per 1 pmol/L)	−0.212	−0.409–0.008	0.032
	Triglycerides (per 1 mmol/L)	−0.235	−0.433–−0.030	0.020

* Statistical significance determined by multivariate regression test adjusted for demographic characteristics of patients; β, regression coefficient; 95%CI, confidence interval; NLR, neutrophil-to-lymphocyte ratio; OSSS-3, Oslo-3 Social Support Scale; iPTH, intact parathyroid hormone.

## Data Availability

The original contributions presented in this study are included in the article; further inquiries can be directed to the corresponding author.
